# Characteristics of the Alternative Phenotype of Microglia/Macrophages and its Modulation in Experimental Gliomas

**DOI:** 10.1371/journal.pone.0023902

**Published:** 2011-08-25

**Authors:** Konrad Gabrusiewicz, Aleksandra Ellert-Miklaszewska, Maciej Lipko, Malgorzata Sielska, Marta Frankowska, Bozena Kaminska

**Affiliations:** Laboratory of Transcription Regulation, Nencki Institute of Experimental Biology, Warsaw, Poland; University of Medicine and Dentistry of New Jersey, United States of America

## Abstract

Microglia (brain resident macrophages) accumulate in malignant gliomas and instead of initiating the anti-tumor response, they switch to a pro-invasive phenotype, support tumor growth, invasion, angiogenesis and immunosuppression by release of cytokines/chemokines and extracellular matrix proteases. Using immunofluorescence and flow cytometry, we demonstrate an early accumulation of activated microglia followed by accumulation of macrophages in experimental murine EGFP-GL261 gliomas. Those cells acquire the alternative phenotype, as evidenced by evaluation of the production of ten pro/anti-inflammatory cytokines and expression profiling of 28 genes in magnetically-sorted CD11b^+^ cells from tumor tissues. Furthermore, we show that infiltration of implanted gliomas by amoeboid, Iba1-positive cells can be reduced by a systematically injected cyclosporine A (CsA) two or eight days after cell inoculation. The up-regulated levels of IL-10 and GM-CSF, increased expression of genes characteristic for the alternative and pro-invasive phenotype (*arg-1*, *mt1-mmp*, *cxcl14*) in glioma-derived CD11b^+^ cells as well as enhanced angiogenesis and tumor growth were reduced in CsA-treated mice. Our findings define for the first time kinetics and biochemical characteristics of glioma-infiltrating microglia/macrophages. Inhibition of the alternative activation of tumor-infiltrating macrophages significantly reduced tumor growth. Thus, blockade of microglia/macrophage infiltration and their pro-invasive functions could be a novel therapeutic strategy in malignant gliomas.

## Introduction

Recent experimental and pre-clinical studies show an important role of tumor-infiltrating macrophages in tumor growth, metastasis and response to cancer treatments [1], [2]. Tumor-associated macrophages are attracted by tumor-released molecules which induce reprogramming/differentiation of macrophages into anti-inflammatory cells known as alternatively activated, in contrast to inflammatory M1-type macrophages. M2-type macrophages from experimental tumors and various types of cancers express arginase-1 (Arg1), IL-10 and transforming growth factor beta 1 (TGF-β1), support tumor invasion, angiogenesis and matrix remodelling [2], [3], [4]. The stromal signals influencing glioma progression are poorly known and are likely distinct from those implicated in non-nervous system cancers. Microglia are brain resident macrophages, which respond to pathogens or injury in the brain. Once activated (e.g. by lipopolysaccharide - LPS or interferon gamma - IFNγ) they are characterized by increased chemotaxis, production of inflammation mediators and cytokines, activation of the respiratory burst, and they become immune effector cells mediating both innate and adaptive responses [5], [6]. Histopathologic and flow cytometry studies of human glioma tissue have shown high intratumoral microglia density which correlates with the grade of malignancy [7], [8], [9]. Microglial cells in tumors do not secrete cytokines critical in developing effective innate immune responses and an anti-tumor immune response is suppressed in glioma patients [9], [10], [11]. The presence of cells positively stained for CD163 and CD204, putative markers for “M2” macrophages [12], as well as myeloid-derived suppressor cell-like cells have been detected in glioma patients [13]. Several glioma-secreted factors have been characterized as promoting microglial chemotaxis: macrophage colony-stimulating factor 1 [12], [14], granulocyte/macrophage colony-stimulating factor [15], hepatocyte growth factor [16] and monocyte chemotactic protein 3 [17]. Immunosuppressive properties of glioma cancer stem cells, producing CSF-1, TGF-β1 and macrophage inhibitory cytokine, and inducing polarisation of recruited macrophages/microglia have been demonstrated [18], [19], [20].

Experimental studies using brain organotypic slices depleted of microglia, genetic models of microglia ablation and microglia-glioma co-cultures pinpoint a pro-invasive role of glioma-infiltrating microglia [21], [22]. Glioma-exposed microglia release metalloproteinase MT1-MMP which activates a latent metalloproteinase 2 (pro-MMP-2) produced by glioma cells that promotes tumor invasion, as was shown using brain slices from MT1-MMP-deficient mice and in a microglia depletion model [23]. Furthermore, microglial cells secrete active TGF-β1, which stimulates glioblastoma invasion [24].

In the present study, we demonstrate that microglia and macrophages accumulate in GL261 experimental gliomas, adapt an anti-inflammatory “M2” phenotype, express arginase-1, IL-10 and MMPs. To interfere with glioma-microglia interactions, we used cyclosporine A (CsA) which has been shown to reduce microglia activation and invasion of glioma cells *in vitro* and in organotypic brain slices [22]. Systemically-applied CsA inhibits microglia/macrophage infiltration, induces cell death of infiltrating cells and blocks expression/activity of enzymes and cytokines, important for the establishment of a pro-invasive phenotype of glioma-infiltrating microglia/macrophages. This results in considerable reduction of tumor growth and angiogenesis. Our results demonstrate that counteracting infiltration of microglia/macrophages into tumor and their differentiation into the “M2” phenotype could be a novel strategy in glioma therapy.

## Materials and Methods

### Ethics statement

This study was conducted under the protocol 857/2008, which was approved by the Local Ethics Committee for Animal Experimentation at the Nencki Institute of Experimental Biology.

### Cell culture and treatment

GL261-glioma cells stably expressing pEGFP-N1 [21] obtained from Prof. Helmut Kettenmann (MDC, Berlin, Germany) were cultured in DMEM with 10% FBS (Gibco, MD, USA) and antibiotics (50 U/ml penicillin, 50 µg/ml streptomycin) in a humidified atmosphere of CO_2_/air (5%/95%) at 37°C (Heraeus, Hanau, Germany). Cyclosporine A (CsA) was purchased from Novartis (Sandimmum, Switzerland) and FK506 from Astellas (Prograf, Astellas Co. Ltd., Ireland). Drugs diluted in PBS were added to cultured cells or intraperitoneally (i.p.) injected to tumor-bearing mice.

Primary cultures of astrocytes were prepared from cerebral cortex of 2-day-old C57BL/6 newborn mice as described previously [25]. Astrocytes were cultured in DMEM with high glucose (GlutaMAX™, Gibco) supplemented with 10% FBS, 100 U/ml penicillin and 100 µg/ml streptomycin.

### In vivo studies

Male C57BL/6 mice (12–16 wk) were anesthetized with an i.p. injection of ketamine (75 mg/kg) and medetomidine (1 mg/kg). EGFP-GL261 glioma cells (8×10^4^ cells in 1 µl of DMEM) were inoculated in a right striatum using 1-µl syringe with a 26-gauge needle in a stereotactic apparatus (Stoelting Co., USA), according to the coordinates (+1.5 mm AP, −1.5 ML). Mice were resuscitated using i.p. administration of atipamazole and analgezed with Tolfedine 4% (4 mg/kg s.c.; Vetoquinol).

After tumor implantation, the animals were housed individually and randomly assigned to treatments: CsA (2 or 10 mg/kg) or FK506 (1 mg/kg) every 2 days starting from the 2nd day after cell implantation (early treatment); every day from 8th day after glioma implantation (postponed treatment); control mice received PBS injections. At day 15th after glioma implantation, the animals were anesthetized, sacrificed and perfused with 4% paraformaldehyde in PBS. The brains were removed, post-fixed for 24 h in the same fixative solution and placed in 30% sucrose in PBS at 4°C. Next, brains were frozen with dry CO_2_ and serial 20-µm-thick coronal sections were collected using a cryostat. Images were acquired using a Leica DM4000B microscope.

### Quantification of tumor volume

Tumor areas in coronal sections were measured using Image Pro-Plus software (v.6.1, Media Cybernetics, Silver Spring, MD) in every second 40-µm brain slice and volumes were calculated according to the Cavalieri principle using algorithm described below.
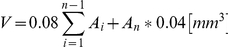
Legend: *V* – volume; *A_i_* – tumor area in a slice; *A_n_* – tumor area in a last slice; 0.08 – constant distance between slices together with slice thickness; 0.04 – thickness of a last slice.

### Immunohistochemistry

Staining with anti-Iba1 antibody was used to detect microglia in the tumor-bearing sections. Sections were treated with a blocking solution (3% normal goat serum, 0.1% Triton X-100 in PBS) for 30 min at room temperature, then stained with the rabbit anti-Iba1 antibody (1∶500, Wako, Japan) overnight at 4°C, followed by 2 h incubation with the Alexa Fluor 647-conjugated goat anti-rabbit antibody (1∶500, Invitrogen).

TUNEL staining was performed on 20-µm free-floating sections with the biggest tumor area according to the manufacturer's protocol (In situ Cell Detection Kit, TMR, Roche Molecular Biochemicals, Indianapolis, IN, USA). Free-floating sections were first incubated in 99% ethanol and 80% acetic acid (2∶1 ratio) at 4°C for 30 min and then for 30 min in the blocking solution: 5% bovine serum albumin, 3% normal goat serum in 0.1% TBS. Subsequently, after brief washing in PBS, sections were incubated with TUNEL reaction mixture for 1 h at 37°C in a dark humid chamber. For the positive control, sections from a sham-operated brain were treated with DNase I (Sigma) prior to labelling. Negative control slices were incubated with a mixture without enzyme. Thereafter, sections were incubated with primary antibodies: rabbit anti-Iba1 (1∶500, Wako, Japan), rabbit anti-GFAP (1∶500, DakoCytomation), rabbit anti-NSE (1∶100, Chemicon) in the blocking solution overnight at 4°C. After washing, slices were stained with Alexa Fluor 647-conjugated goat anti-rabbit antibody (Invitrogen) in the blocking solution at 1∶500 dilution for 2 h at 25°C. Incubation was followed by labeling with DAPI (Sigma), dehydration in alcohol and mounting with DPX Mountant for histology (Fluka). Brain sections were examined under a fluorescent Leica DM4000B microscope and a confocal microscope (TCS SP2, Leica). Image Pro-Plus software was used for quantification of TUNEL-positive cells.

### Measurement of pro- and anti-inflammatory cytokines in brain extracts

Measurement of pro- and anti-inflammatory cytokines was performed in whole brain extracts from naïve and tumor-bearing mice; 2 µl of LPS (1 mg/ml, Sigma) was injected intracerebrally as a positive control. Naïve, LPS-treated (6 h post-injection) and tumor-bearing mice (14 d after glioma implantation) were anesthetized and transcardially perfused with ice-cold PBS. Ipsilateral hemispheres were quickly collected and tissues were homogenized in 10× volume lysis buffer (50 mmol/L Tris-HCl, pH 7.4, 150 mmol/L NaCl, 1% Nonidet P-40, 0.1% SDS), with protease inhibitors (2 µg/ml leupeptin, 2 µg/ml aprotinin, 1 mmol/L PMSF) on ice using a homogenizer (Ultra-Turrax T8, Ika-Werke). After centrifugation (10 000 g) for 10 min at 4°C, the supernatants were collected and stored at −80°C. The levels of cytokines were measured using the FlowCytomix Mouse Th1/Th2 10plex assay (Bender MedSystems, Vienna, Austria). Briefly, tissue extracts were incubated with a mixture of antibodies-coated fluorescent beads characterized by different size and fluorescent signature. Next, a biotin-conjugated antibody mix followed by a streptavidin-PE solution were added to the beads to quantify the captured analyte. Samples were run on a flow cytometer (FACScan, Becton Dickinson), analyzed with FlowCytomix Pro 2.2 Software (Bender MedSystems), and referred to a standard curve. Results were expressed as pg/ml for each cytokine.

### Isolation of CD11b-positive cells and flow cytometry

Naïve, sham-operated and tumor-bearing mice were anesthetized and decapitated. Brains were removed, brain tissues were cut into small pieces and single-cell suspension was achieved by enzymatic digestion using the Neural Tissue Dissociation Kit (Papain) from Miltenyi Biotec. The tissue was further mechanically dissociated using a wide-tipped, fire-polished Pasteur pipette and the suspension was applied to a 30 µm cell strainer. Cells were processed immediately for MASC MicroBead separation. The CD11b-positive cells were magnetically labelled with CD11b MicroBeads. The cell suspension was loaded onto a MACS Column (Miltenyi Biotec) placed in the magnetic field of a MACS Separator and a negative fraction was collected. After removing the magnetic field, CD11b-positive cells were eluted as a positive fraction. Additionally, cells were stained with CD11b-PE and CD45 PerCP-Cy5.5 antibodies (BD Pharmingen). IL-10 expression on microglia/macrophages was determined by a staining of CD11b^+^ cells with Alexa Fluor 647 labelled IL-10 antibody (1∶20, Biolegend). Propidium iodide (PI, Sigma) was added (1 µg/ml) to exclude debris and dead cells from analysis. All live CD11b-positive cells were assessed by flow cytometry (FACSCalibur). Data were acquired and analyzed using CellQuest software.

### RNA isolation and quantification of gene expression

Total RNA from sorted CD11b^+^ cells was isolated with RNeasy Mini Kit (Qiagen, Hilden, Germany). The quality and yield of RNAs were verified using the 2100 Bioanalyzer (Agilent Technologies, Santa Clara, CA). First-strand cDNA was synthesized from 1 µg of total RNA (DNase-treated) in a 20 µl reverse transcriptase reaction mixture. TaqMan Express Plates (Applied Biosystems) with specific primers and FAM labelled probe sets were employed for determination of *arg-1*, *ccl7*, *chi3l1*, *chi3l3 (ym1)*, *c-myc*, *cox-2*, *cx3cr1*, *cxcl14*, *fizz1 (retnla)*, *hif-1α*, *ifn-β1*, *il-10*, *il-12α*, *il-17α*, *il-1β*, *inos*, *irf-7*, *kcnk10*, *mt1-mmp*, *mmp2*, *mmp9*, *smad6*, *smad7*, *socs2*, *stat1*, *stat3*, *tgf-β1* and *tnfsf10* expression; 18s rRNA was used as an internal reference gene. The thermal cycling conditions were: 10 min at 95°C and 40 cycles of 95°C for 15 sec and 1 min at 60°C for annealing and extension. The expression of *m-csf* and *gm-csf* in GL261 glioma cells and primary astrocytes was evaluated using quantitative real-time PCR. Reaction volume (20 µl) consisted of cDNA equivalent to 50 ng RNA, 1× SYBR Green PCR master mix (Applied Biosystems) and 0.9 µM of each primer. The thermal cycling conditions were as follows: 50°C for 2 min, 95°C for 10 min, followed by 40 cycles of 15 s at 95°C for denaturation and 1 min. at 60°C for annealing and extension. Relative quantification of gene expression was determined using the comparative CT method.

### Measurement of arginase activity

Arginase activity was determined in brain tissue extracts using a colorimetric method based on the conversion of L-arginine to L-ornithine as described [26]. Optical density of ornithine-ninhydrin complexes formed in the samples was measured using a microplate reader at 515 nm. Arginase activity was expressed as U/mg protein (1 Unit = 1 µmol of L-arginine to ornithine per minute at pH 9.5 and 37°C).

### Evaluation of MMP-2 activity by gelatin zymography and fluorescent assay

Tissue extracts (70 µg protein) were resolved by SDS-PAGE on 8% gel containing 2 mg/ml of gelatin (Sigma), 380 mM TRIS-HCl (pH 8.8), 0.1% SDS, 0.1% ammonium persulfate and 0.6 µl/ml of TEMED (tetramethylethylenediamine, Sigma). After electrophoresis, the gel was rinsed twice in 2.5% Triton X-100 at room temperature and renaturated in a buffer containing 50 mM Tris-HCl pH 7.6, 10 mM CaCl_2_, 1 µM ZnCl_2_, 1% Triton X-100 and 0.02% sodium azide (at 37°C, 100 rpm). After 72 hours the gel was stained with Coomasie brilliant blue. MMP-2 activity in tissue extracts was quantified by measuring the fluorescence released by cleavage of DQ-gelatin (Invitrogen). The extracts were incubated with 100 µg/ml of FITC-gelatin in 0.5 M Tris-HCl, 1.5 M NaCl, 50 mM CaCl_2_, 2 mM sodium azide, pH 7.6, for 24 hours at 37°C. Fluorescence intensity was measured in a microplate reader (excitation 485 nm, emission 530 nm).

### Statistical analysis

The results were expressed as means ± standard error of the mean (SEM). Statistical analyses were performed using a Statistica software (ver. 7.1 StatSoft. Inc, OK, USA). Statistical significance was determined by U-Mann-Whitney and ANOVA tests.

## Results

### Activated microglia and blood derived macrophages accumulate in the tumor

**Figure 1:**
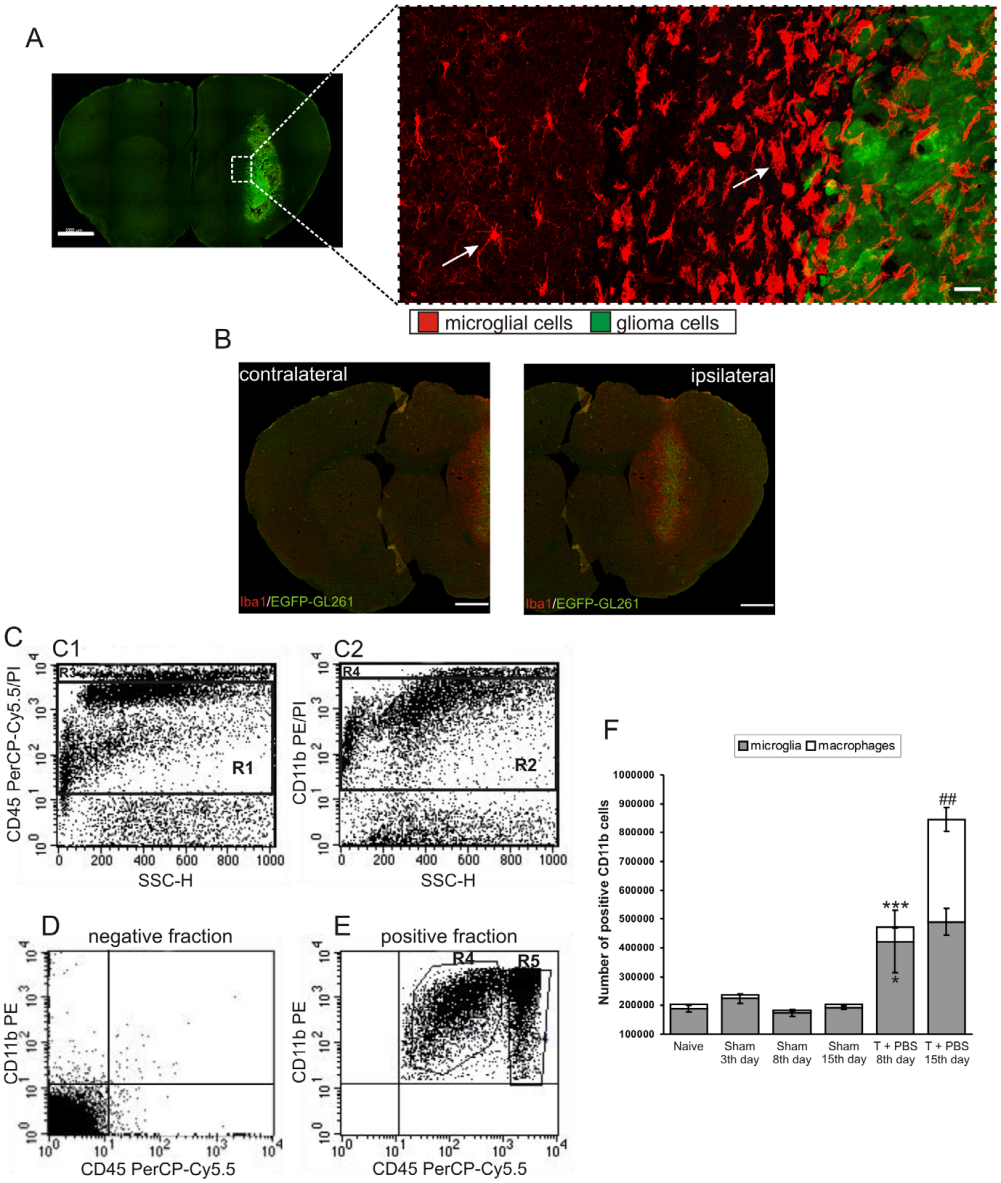
Accumulation and activation of microglia/macrophages in experimental glioma. A. Representative confocal images of brain sections 15 days after implantation of pEGFP-N1 GL261 cells into the striatum of C57BL/6 mice. Note the infiltration and morphological transformation of glioma-infiltrating Iba1^+^ cells. Scale bar: left image – 1000 µm, right image – 20 µm. B. Contralateral and ipsilateral hemisphere from tumor-bearing brain 15 days after injection of pEGFP-N1 GL261 cells. Images showed merged Iba1 and EGFP fluorescence. Scale bar = 750 µm. C. Microglia/macrophages were separated using a magnetic-bead-conjugated anti-CD11b antibody and stained with CD45 PerCP-Cy5.5 and CD11b PE prior to FACS acquisition. Propidium iodide staining was performed to eliminate necrotic/apoptotic cells (Gate R3, R4) and viable cells were gated (Gate R1; **B1**, Gate R2; **B2**). D. Efficiency of CD11b-positive cells separation in the negative fraction (CD11b-negative cells) from each sample was controlled. E. Representative dot plots for microglia (Gate R4, CD11b^+^/CD45^low^) and macrophages (Gate R5, CD11b^+^/CD45^high^) from tumor-bearing hemispheres. F. Kinetics of microglia/macrophage influx into tumor tissue. CD11b^+^ cells separated from the brains of naïve, sham operated and tumor-bearing mice at day 3, 8 or 15 after implantation (n = 4/group) were sorted according to CD45 expression. Each bar represents the mean ± SEM; ^***^ *p*<0.001, ^*^ *p*<0.05 tumor-bearing mice at 8th day versus naïve mice; ^##^ *p*<0.01 tumor-bearing mice at day 15 versus day 8.

GL261-glioma cells strongly attract Iba1-positive microglia/macrophages and cause their morphological transformation (Fig. 1A). Only a few ramified Iba1-positive cells were detected in the intact brain. Numerous amoeboid, Iba1-positive cells accumulated within and around the implanted glioma cells, while ramified microglia, with thin branching processes and a small cell body were detected in the tumor-free parenchyma (Fig. 1A). Interestingly, the cells in the close vicinity of tumor were evidently more activated and amoeboid than the cells located distantly. Microglia specific staining was mostly localized in the ipsilateral hemisphere (Fig. 1B).

Differences in the surface expression of CD11b and CD45 antigens on microglia (CD11b^+^/CD45^low^) and blood-derived macrophages (CD11b^+^/CD45^high^) isolated from tumor tissue permitted distinction of the two populations by flow cytometry [27]. We determined the relative contribution of resident and blood-derived cells to the Iba1-positive population within the tumor. Since microglia/macrophages are the major CD11b-expressing cell population in the brain, a magnetic-bead-conjugated anti-CD11b antibody was used to separate these cells from tumor-bearing hemispheres. Propidium iodide was added to the samples to exclude debris and dead cells. All live CD11b/CD45-positive cells were acquired and analyzed using CellQuest software (Fig. 1C). Quadrant gates were drawn on the negative fraction containing mostly CD45^−^/CD11b^−^ cells (Fig. 1D) and detection of only few CD11b^+^/CD45^+^ cells, confirmed a sensitive separation. Microglia and macrophages (Fig. 1E) were identified according to their specific surface antigen expression profile as CD11b^+^/CD45^low^ and CD11b^+^/CD45^high^ populations, respectively. In naïve, microglia constituted the prevalent population (93%) of CD11b^+^ cells in the brain and the number of microglia/macrophages did not change in sham-operated animals injected with medium alone (Fig. 1F). The number of microglia raised significantly from the 8th day, while the number of macrophages increased at the 15th day after tumor implantation (Fig. 1F).

**Figure 2:**
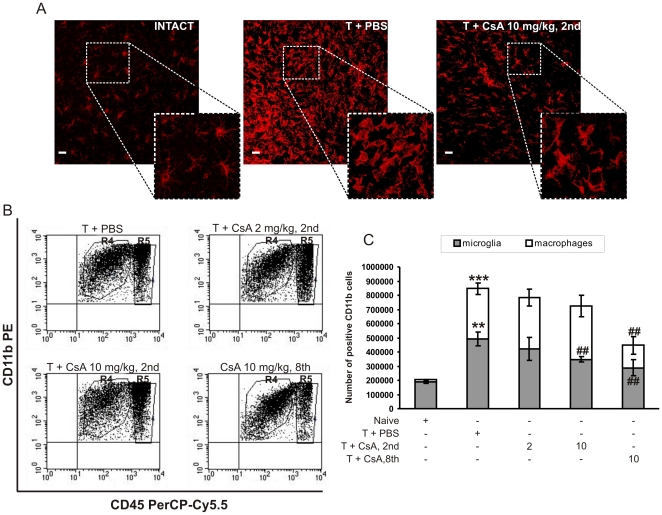
Influx of microglia/macrophages into the tumor is blocked by CsA. A. Representative confocal images of Iba1 staining in intact brain tissue, tumor-bearing brain slices from mice treated with PBS or CsA. Scale bar = 20 µm. B–C. Quantification of microglia and blood-derived macrophages in naïve, tumor-bearing and CsA-treated mice (4 per group). Each bar represents the mean ± SEM. ^***^ *p*<0.001, ^**^ *p*<0.01 tumor-bearing versus naïve mice; ^##^ *p*<0.01, CsA-treated versus PBS-treated tumor-bearing mice.

CsA effectively impairs the pro-invasive activity of microglia *in vitro* and in organotypic brain slices [22]. As kinetic studies indicated different phases of microglia and macrophage accumulation, CsA was injected starting from the 2nd or 8th day after tumor implantation. Systemic treatment with CsA (2 and 10 mg/kg), reduced the abundance of Iba1-positive cells (Fig. 2A). These cells were only moderately enlarged and less activated in CsA-treated mice as compared to PBS-treated mice. Quantification of tumor-infiltrating microglia/macrophages revealed a ∼2.6-fold increase in the number of microglia and a ∼30-fold increase in the number of macrophages 15 days after tumor implantation. Early treatment with 10 mg/kg CsA reduced the number of infiltrating microglia by 27%, while postponed administration of CsA strongly reduced infiltration of both microglia (by ∼47%) and macrophages (by 71%) (Fig. 2B–C). Immunofluorescence studies and quantification of glioma-infiltrating CD11b^+^ cells clearly demonstrate inhibition of accumulation of these cells by CsA in experimental gliomas.

**Figure 3:**
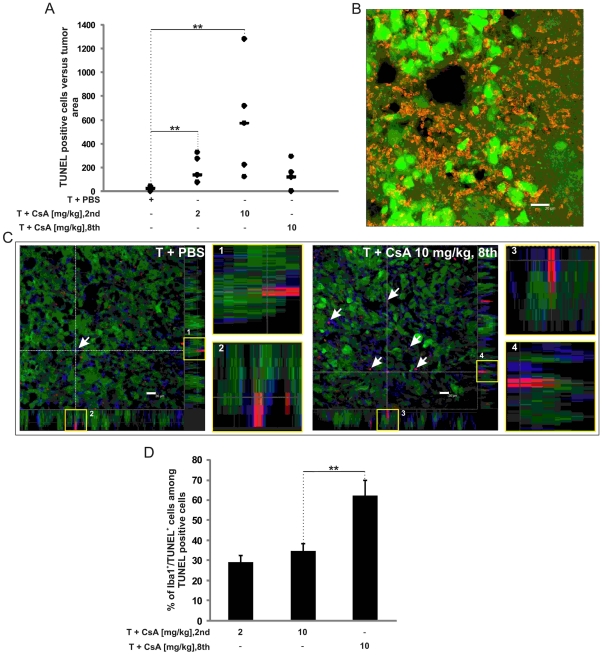
CsA induces cell death within tumor-associated microglia/macrophages. A. Quantification of the number of TUNEL-positive cells per 1 mm2 of tumor area in saline and CsA-treated mice was counted using Image Pro-Plus software. The results show the median of 5–6 mice in each group. A statistical significance was determined by U-Mann-Whitney test; **p<0.01. B. Detection of DNA fragmentation *in situ* in EGFP-N1 GL261 tumour bearing brain section visualised with TUNEL staining (red fluorescence) by confocal microscopy. Scale bar = 20 µm. C. Identification of TUNEL-positive cells (red) in tumour-bearing slices. Sections were subjected to TUNEL reaction and stained for Iba1 (microglia/macrophages). Images were acquired using confocal microscopy and Z-stack projection was performed. Representative Z-stack images (1–4) of saline- and CsA-treated mice (10 mg/kg, postponed treatment) demonstrate a co-localization of TUNEL-positive cells (red) and Iba1-positive cells (blue). Scale bar = 20 µm. D. Quantification of Iba+/TUNEL+ cells among TUNEL-positive cells. The results show the mean ± SD (4–6 mice per group); **p<0.01 significant change between postponed (10 mg/kg) and early treated mice (10 mg/kg).

Detection of DNA fragmentation *in situ* by TUNEL staining on brain slices revealed an increase in the number of apoptotic cells after CsA treatment (Fig. 3A). Confocal microscopy analysis showed that most of the TUNEL-positive cells (in red) in CsA-treated animals did not co-localize with the implanted tumor cells (in green) (Fig. 3B). Staining with antibodies against GFAP (glial fibrillary acidic protein, a marker for astrocytes) and NSE (neuronal specific enolase, a marker for neurons) showed no co-localization of neuronal/astroglial markers with TUNEL positive cells (not shown). However, some TUNEL-positive cells (in red) co-localized with Iba-1 staining (in blue) (Fig. 3C), that is consistent with the induction of cell death mostly among glioma-infiltrating activated microglia/macrophages in CsA-treated mice. Quantification of Iba1^+^/TUNEL^+^ cells among TUNEL-positive cells inside the tumor revealed an increase in the number of double-positive cells after delayed CsA treatment (Fig. 3D).

### Tumor-infiltrating microglia/macrophages do not produce pro-inflammatory cytokines

**Figure 4:**
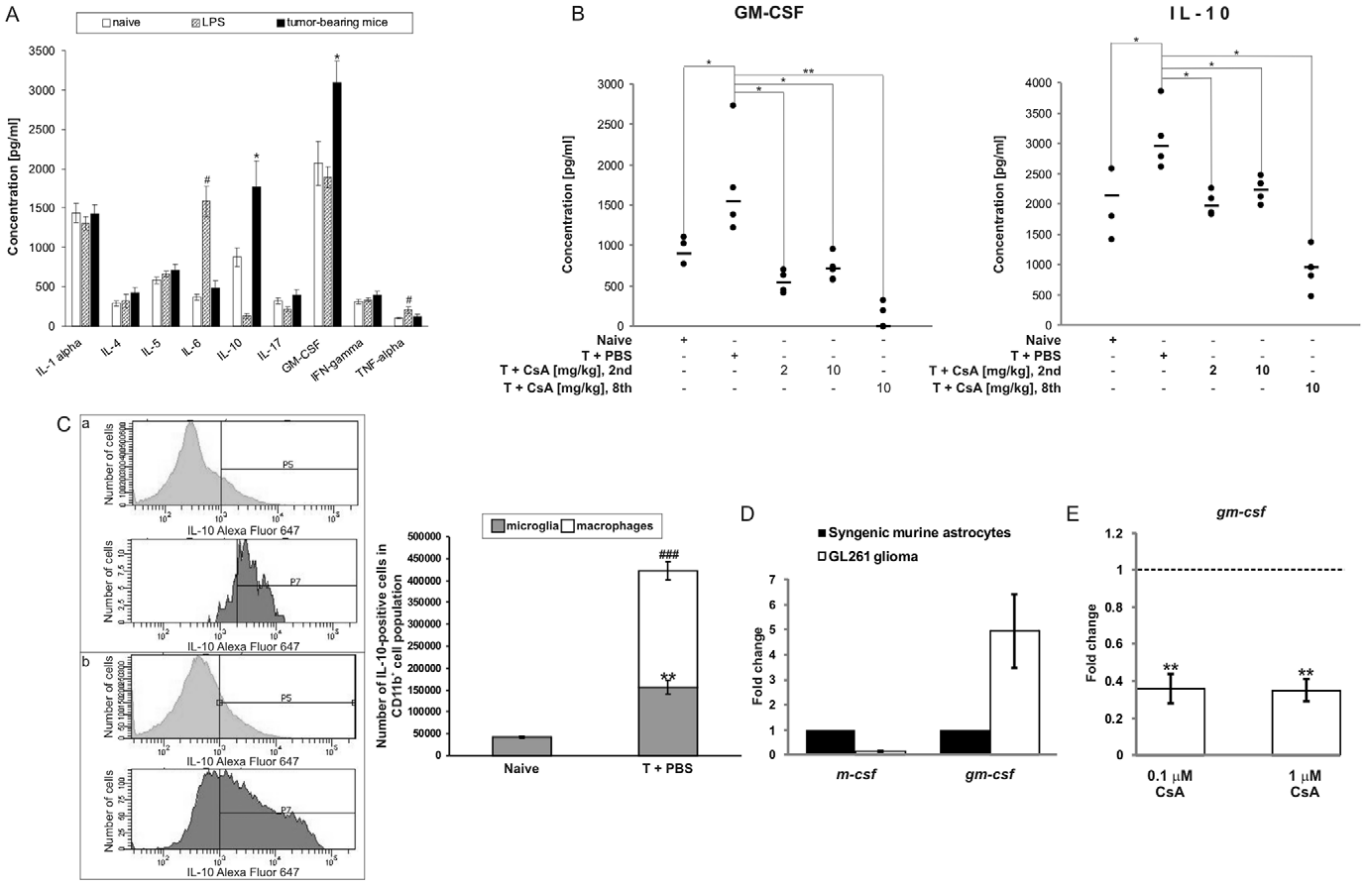
Cytokine profile in glioma-bearing brains. A. The levels of ten pro/anti-inflammatory cytokines were determined by flow cytometry in protein extracts isolated from hemispheres of naïve, LPS-injected and tumor-bearing mice. The results show the means ± SEM (n = 5 per group); ^#^ *p*<0.05 significant change between LPS-treated v. naïve mice; ^*^ *p*<0.05 significant difference between tumor-bearing v. naïve mice. B. Elevated levels of IL-10 and GM-CSF detected in tumors are reduced by CsA treatment. Each dot represents an individual animal; a horizontal line represents a mean of each group; ^*^ *p*<0.05; ^**^ *p*<0.01. C. Expression of IL-10 in glioma-infiltrating microglia and macrophages. Left panel: expression of IL-10 on sorted CD11b^+^ cells (50,000 cells) determined by flow cytometry with the anti-IL-10 antibody conjugated to Alexa Fluor647. Representative histograms of IL-10 detection in microglia (light grey) and macrophages (dark grey) cells from naïve (a) and tumor-bearing (b) brains. Right panel: quantification of microglia/macrophages expressing IL-10 in naïve and tumor-bearing brains (means ± SEM, 3 animals per group); significant increase of IL-10-positive microglia (** *p*<0.01) and macrophages (^###^ *p*<0.001) in tumor-bearing brains. D. Quantification of *gm-csf* and *m-csf* expression in GL261 glioma cells and non-transformed astrocytes (means ± SD from 3 experiments). E. Quantitative evaluation of *gm-csf* mRNA expression in GL261 glioma cells exposed to 0.1 and 1 µM CsA for 24 hours (means ± SD from two experiments with three or four replicates per condition) compared to untreated control cells. Statistical analysis was done by Student *t* test, ** *p*<0.01.

To understand the functional relevance of glioma infiltration by immune cells, we determined the levels of pro- and anti-inflammatory cytokines in whole brain tissue extracts using a multiplex 10 Th1/Th2 cytokine assay. An intracerebral LPS injection up-regulated TNFα and IL-6 levels, but reduced IL-10, IL-17 and GM-CSF levels. In tumor-bearing mice only IL-10 and GM-CSF levels were significantly elevated when compared to naïve mice (Fig. 4A). Interestingly, CsA treatment strongly reduced IL-10 and GM-CSF production, with the postponed treatment being the most effective (Fig. 4B). Flow cytometric analysis of magnetically sorted cells revealed that the IL-10 protein is expressed mostly in CD11b^+^/CD45^high^ macrophages (Fig. 4C). The level of *gm-csf* expression, but not *m-csf*, was significantly higher in GL261 glioma cells than in non-transformed murine astrocytes (Fig. 4D). Treatment with 0.1 and 1 µM CsA (doses corresponding to *in vivo* blood concentrations) leads to downregulation of *gm-csf* mRNA level in GL261 glioma cells (Fig. 4E).

### Gene expression in glioma-infiltrating microglia/macrophages

**Figure 5:**
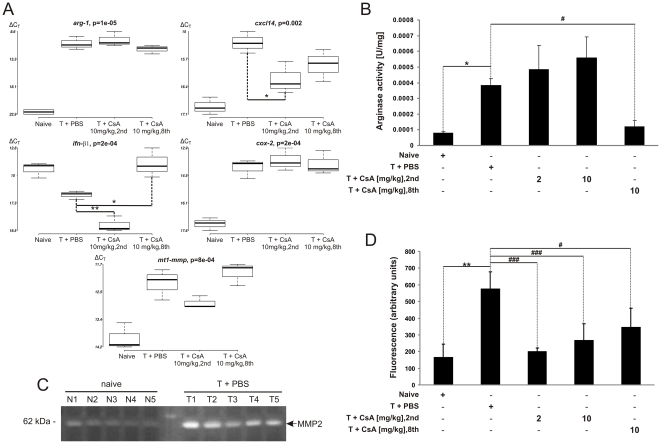
Alterations of gene expression in infiltrating microglia/macrophages and intracranial gliomas are modulated by CsA. A. Gene expression in magnetically sorted CD11b^+^ cells from tumor-bearing and naïve brains was determined by qPCR. Expression of five genes was significantly altered in CD11b^+^ cells: *arg-1 (p = 0.000003)*; *cxcl14 (p = 0.0001)*; *ifn-β1 (p = 0.0002)*; *cox-2 (p = 0.000002)*; *mt1-mmp (p = 0.00002)*; n = 5 animals per group; ^*^ *p*<0.05, ^**^ *p*<0.01. The middle line represents the median value. Lower ΔC_T_ are consistent with higher gene expression. B. Quantification of arginase activity in brain tissue extracts from naïve and tumor-bearing mice treated either with PBS or CsA. Results represent the mean ± SEM of 4–5 mice; ^*^ *p*<0.05, tumor-bearing versus tumor-free hemispheres; ^#^ *p*<0.05, CsA (10 mg/kg, 8th) versus PBS-treated, tumor-bearing mice. C. MMP-2 activity in proteins extracts from the brains of naïve (N1–5) and tumor-bearing mice (T1–5) determined by gel zymography. Active MMP-2 detected as a prominent band at 62 kDa. D. Quantification of MMP-2 activity using the cleavage of fluorescent DQ-gelatin substrate; means ± SEM of 4–6 mice; ^**^ *p*<0.01, tumor-bearing versus naïve brain extracts; ^###^ *p*<0.001, ^#^ *p*<0.05, CsA- versus PBS-treated tumor-bearing mice.

We analysed the expression of 28 genes, putatively characterizing the M1- or M2-type of macrophages (Table 1), in magnetically sorted CD11b^+^ cells from naïve and tumor-bearing mice. The expression of five genes: *arg-1*, *cxcl14*, *ifn-β1*, *cox-2*, *mt1-mmp* significantly differed in CD11b^+^ cells from tumor comparing to naïve mice. Up-regulation of *arg-1*, *cxcl14*, *mt1-mmp* and *cox-2* expression and down-regulation *ifn-β1* in tumor CD11b^+^ cells was observed (Fig. 5A). Early treatment with CsA decreased *ifn-β1*, *cxcl14* and *mt1-mmp* expression (*p*<0.05) in tumor CD11b^+^ cells compared to controls, and delayed CsA administration decreased *arg-1* and *cxcl14* expression. Up-regulated expression of *cox-2* in CD11b^+^ cells from gliomas remained unaffected by CsA. However, the expression of *il-1β* was up-regulated in CD11b^+^ cells from gliomas, this increase was smaller in comparison to 25-fold increase in the *il-1β* mRNA level in CD11b^+^ cells from LPS-injected brains (not shown).

**Table 1:** Quantification of selected M1/M2 phenotype-associated gene expression in CD11b^+^ cells isolated from naïve and tumor-bearing mice.

Genes	CD11b^+^ cells from glioma	
	Fold change	p-value
***arg-1***	**3734.42**	**7.00E-04**
*ccl7*	0.72	0.3951
*chi3l1*	1.31	0.7441
*chi3l3 (ym1)*	7.24	0.0586
***c-myc***	**0.27**	**0.0478**
*cx3cr1*	0.14	0.0643
*cxcl14*	3.21	0.0016
*hif-1α*	1.57	0.4294
***ifn-β1***	**0.24**	**0.0137**
*il-10*	0.75	0.6344
*il-12α*	14.83	NA
*il-17α*	8.24	NA
***il1-β***	**11.09**	**1.00E-04**
***irf-7***	**30.11**	**9.00E-04**
*kcnk10*	4.63	NA
*mmp-9*	0.78	0.337
***mt1-mmp***	**3.21**	**0.0099**
*mmp-2*	0.56	0.699
*inos*	88.82	0.085
***cox-2***	**9.57**	**0.018**
*fizz1 (retnla)*	0.36	0.0731
*smad-6*	1.25	0.6346
*smad-7*	0.26	0.2283
*socs-2*	5.89	0.0671
***stat-1***	**10.38**	**2.00E-05**
*stat-3*	0.9	0.5458
*tgf-β1*	0.68	0.1778
*tnfsf10*	2.11	0.2265

Gene expression was analyzed by real-time PCR and the results are presented as fold changes of CD11b^+^ cells isolated from tumor brains versus those from naïve brain tissue. Numbers corresponding to the significantly changed genes (t-test generated p-value<0.05) are marked in bold; NA - not available.

We assessed total gelatinase activity in the tumor extracts using gelatin zymography. Only MMP-2 activity was clearly detected in extracts from tumor-bearing brains, and it was higher than in extracts from tumor-free brains (Fig. 5C). Changes in MMP-2 activity were confirmed using a fluorescein-conjugated DQ gelatin assay. Following CsA treatment, MMP-2 gelatinolytic activity was significantly reduced by CsA treatment, particularly after early treatment CsA (Fig. 5D). The level of *arg-1* mRNA was elevated in tumor-derived CD11b^+^ cells, thus we measured arginase activity in brain extracts. Figure 5B shows increased arginase activity in extracts from tumor-bearing brains in comparison to tumor-free tissue. Only the delayed treatment diminished significantly arginase activity.

### CsA treatment strongly impairs the growth of intracranial gliomas

**Figure 6:**
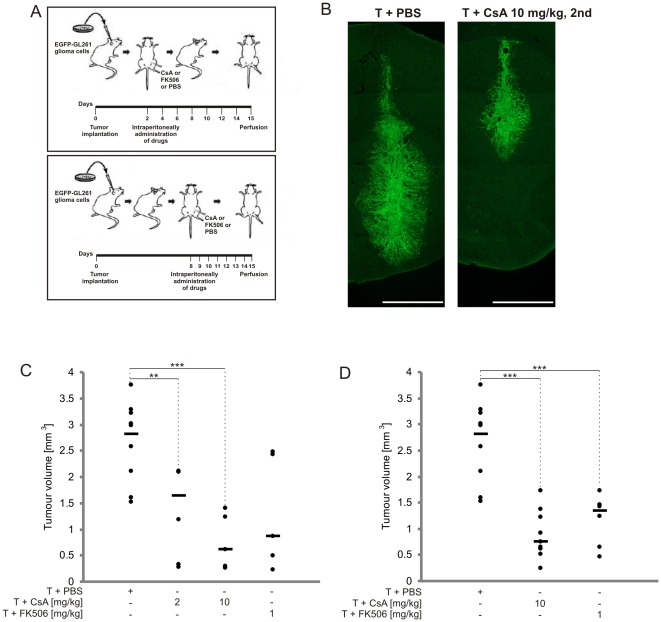
Reduction of tumor growth in CsA-treated animals. A. Scheme of experiment. B. Representative images of EGFP-GL261 tumors in mice treated with PBS or 10 mg/kg CsA, 2nd; scale bar = 1 mm. C–D. Quantification of tumor volumes in PBS, CsA-treated or FK506-treated mice after early (C) or postponed (D) treatment. Results for individual animals are presented; the bold line represents the median of 5–10 mice in each group. ^**^ *p*<0.01, ^***^ *p*<0.001, tumor volumes in drug-treated versus control mice.

As accumulation of tumor-associated CD11b^+^ cells with some features of the M2 phenotype was reduced by CsA treatment, we determined if CsA and FK506 (similarly acting drug, more effectively crossing the blood brain barrier) would affect tumor growth. Drugs were injected i.p., every two day starting from the 2nd day or every day from the 8th day after glioma inoculation (Fig. 6A). CsA treatment strongly reduced tumor growth *in vivo* (Fig. 6B). Quantification of tumor volumes in CsA- and FK506-treated mice showed significantly smaller tumors when compared to control mice. After early treatment with either 2 or 10 mg/kg CsA, tumor volume was reduced by 59% (1.15±0.36 mm^3^) and ∼78% (0.63±0.24 mm^3^), respectively, versus an average tumor volume of 2.83±0.23 mm^3^ in control mice. In FK506-treated mice, tumor reduction reached ∼69% (0.88±0.48 mm^3^) (Fig. 6C). When the treatment was postponed to the 8th day, only the higher dose of CsA (10 mg/kg) was effective in reducing tumor volume by ∼73% (0.76±0.16 mm^3^). FK506 treatment diminished tumor volume by ∼52% (1.35±0.20 mm^3^) (Fig. 6D).

## Discussion

The concept that macrophages play instructive roles in tumor growth by regulating cell motility, invasion, angiogenesis and immunomodulation has gained great attention over last years. [2], [28], [29]. Tumor-derived cytokines such as IL-4, IL-10, TGF-β, and M-CSF are believed to polarise tumor-infiltrating macrophages towards an M2 anti-inflammatory phenotype [30], [31]. Mechanisms of accumulation and a role of microglia/macrophages in glioma pathobiology are still poorly understood and controversial. Several experimental studies demonstrated that microglia contribute to glioma progression by secreting growth factors, angiogenic molecules, extracellular matrix–degrading enzymes, and immunosuppressive factors [21], [22], [23], [24]. However, Galarneau et al. showed that ablation of CD11b cells (ganciclovir-treated B6 mice expressing a mutant form of HSV-1 TK^mt-30^ under CD11b promoter) increases glioma growth [32], a recent study demonstrated that ablation of CD11b *in vivo* (ganciclovir-treated CD11b-HSVTK mice) decreases tumor size and improves survival [33].

We demonstrated that Iba1-positive cells infiltrate implanted GL261 gliomas and close distance interactions result in amoeboid transformation of these cells. Our analysis of sorted CD11b^+^ cells followed by CD11b/CD45 staining shows the increase in a number of microglia (CD11b^+^/CD45^low^) and a surprisingly high infiltration of tumor tissue by blood-derived macrophages (CD11b^+^/CD45^high^). Kinetics of changes in the number of microglia and macrophages infiltrating gliomas showed early accumulation of microglia in the first week, followed by accumulation of macrophages afterwards.

Amongst ten analyzed pro/anti-inflammatory cytokines, only IL-10 and GM-CSF levels were elevated in the tumor-bearing brains comparing to naïve mice. Flow cytometry analysis of magnetically-sorted CD11b^+^ cells demonstrates that IL-10 is produced mostly by infiltrating macrophages. The expression of *gm-csf* was 5 times higher in GL261 glioma cells than in cultured murine astrocytes; therefore, these cells are likely a source of newly synthesized cytokine. The relevance of this finding for human pathology is unclear, because although human astrocytoma and glioblastoma cell lines produce GM-CSF, no evidence of its production by glioblastoma cells was found *in vivo* [34]. Our findings suggest that GM-CSF and IL-10 could be important cytokines for establishment of a pro-invasive phenotype of glioma-infiltrating microglia/macrophages.

The present study shows for the first time the expression of putative markers of the M2 phenotype in CD11b^+^ cells infiltrating gliomas. Out of many markers, Arginase 1 is the most consistent. Arginase 1 catalases arginine hydrolysis to urea and ornithine, and competes for its substrate with inducible nitric oxide synthase (iNOS) in IFN-γ-stimulated macrophages. The macrophagic expression of Arg-1 is tightly regulated by exogenous stimuli such as IL-4 and IL-13 [35]. L-arginine depletion due to extensive myeloid arginase activity may suppress T cell immune responses [36]. Expression of iNos and Arg-1 define classically and alternatively activated macrophages in the context of Th2-polarised immune responses [37], [38]. Enhanced expression of Arg-1 associated with TAMs was found in 3LL murine lung carcinoma [39], in the human papillomavirus E6/E7-expressing murine tumors [40] and in CD11b^+^/CD14^−^ myeloid cells from renal carcinoma patients [41]. Cyclooxygenase-2 (COX-2) expression and prostaglandins (PGE) have been implicated in the development of many cancers [42] serving as pro-inflammatory mediators under some conditions, but having anti-inflammatory and immunosuppressive properties under other conditions [43]. Stimulation of PGE2 signalling in dendritic cells facilitates their migration and maturation, while in T cells potently suppresses activation and proliferation [44]. The observed up-regulation of *cox2* mRNA in CD11b^+^ cells corroborates with a study showing the macrophagic expression of COX-2 and enhanced production of PGE1 by glioma-derived factors [45]. A chemokine CXCL14 was identified as a chemoattractant for monocytes [46], and can induce dendritic cell migration and maturation [47], [48]. The enhanced expression of *cxcl14* in glioma-infiltrating CD11b^+^ cells could be involved in macrophage infiltration or their differentiation. Down-regulation of *cxcl14* expression in CsA-treated mice correlates with reduced CD11b^+^ cell accumulation.

The development of a tumor vasculature is a crucial step for the survival and metastasis of malignant tumors. Accumulation of tumor-associated macrophages correlates with the formation of a blood vessel network and the transition to malignancy in the MMTV-PyMT experimental breast cancer in mice. Depleting macrophages in tumors in CSF-1 null mice (*Csf1^op^*) delayed the angiogenic switch and malignant transition, whereas restoration of macrophages rescued the vessel phenotype in these tumors [29]. High-grade gliomas demonstrate the highest levels of tumor angiogenesis when compared to non-neural solid cancers. Numbers of infiltrating macrophages have been found to correlate with small vessel density and tumor grade [49]. We observed an increase in microglia/macrophage infiltration and the development of a dense vessel network in implanted gliomas (not shown). Furthermore, early treatment with CsA inhibited vessel network development in tumor-bearing mice. Evaluation of implanted GL261 mouse gliomas by MR microimaging indicated blood-brain barrier disruption already in early-stage of tumors (1–2 week) [50]. Thus intraperitoneally administered CsA can easily penetrate brain parenchyma.

Systemic delivery of CsA at doses of 2 or 10 mg/kg significantly attenuate tumor growth in C57BL/6 mice; weaker effects were observed after the treatment with FK506. Tumor volumes were decreased even when the CsA injection was postponed until the 8th day after glioma implantation. High expression of Arg-1 and IL-10 with low expression of inflammatory cytokines suggested differentiation of CD11b^+^ cells into the M2 phenotype. The reduction of *cxcl14* expression in CD11b^+^ cells, decrease of GM-CSF and IL-10 levels in CsA-treated mice suggest that CsA down-regulates the production of factors crucial for accumulation and alternative activation of microglia/macrophages. Moreover, CsA treatment resulted in the reduced expression of *mt1-mmp* in glioma-infiltrating CD11b^+^ and overall down-regulation of the MMP-2 activity. Previous studies showed an important role of microglia-derived MT1-MMP in regulation of MMP-2 activity [21], [23].

Altogether, we demonstrate that resident microglia and blood-derived macrophages contribute to a pool of glioma-infiltrating immune cells, regulate tumor angiogenesis and invasion, which are essential for glioma progression. Induction of cell death in tumor-infiltrating microglia/macrophages considerably reduced a pool of TAM and might cause an attenuation of tumor growth. Postponed treatment with CsA seems to be more effective than early treatment because it affects already accumulated microglial cells and delays attraction of blood-derived macrophages. Despite of developments of new therapeutics, glioblastomas are difficult to treat due to frequent dysfunctions of tumor suppressors and oncogenes; the mean survival of patients with glioblastomas ranges between 1–2 years [51], [52]. Counteracting of brain macrophage accumulation and activation should be taken into account when considering the development of more effective therapy against malignant gliomas.
